# Why is teaching math to my child difficult? Experiences from mothers of children with autism spectrum

**DOI:** 10.3389/fpsyg.2026.1918249

**Published:** 2026-09-23

**Authors:** Ayşegül Akıncı Coşgun, Feyza Ucar Cabuk, Adile Emel Sardohan Yildirim

**Affiliations:** 1 Department of Preschool Education, Faculty of Education, Aksaray University, Aksaray, Türkiye; 2 Department of Preschool Education, Faculty of Education, Mersin University, Mersin, Türkiye; 3 Department of Special Education, Faculty of Education, Akdeniz University, Antalya, Türkiye

**Keywords:** autism, early childhood, early mathematics, home math environment, mother experience

## Abstract

**Introduction:**

Children’s mathematics learning processes are significantly shaped by parental involvement in their home environments. In the context of children with autism, this process takes on a unique structure owing to individual characteristics and learning dynamics. However, the literature offers limited findings regarding how parents of children with autism structure mathematics instruction and the strategies they use. This study aimed to examine the perceptions of parents of children with autism regarding mathematics instruction and the teaching approaches and strategies they use at home to teach mathematics. In addition, it addresses how autism-specific characteristics affect the mathematics teaching process.

**Methods:**

This study used a qualitative research approach to examine mothers’ experiences and perceptions of the processes of learning and teaching mathematics at home for children with autism.

**Results:**

The findings indicate that parents mostly teach mathematics in the context of daily life, using natural and interaction-based approaches. Game-based practices, asking questions, and using concrete materials were the strategies most frequently employed by the participants. Moreover, mothers perceived certain autism-related characteristics as influencing the mathematics teaching process at home.

**Conclusion:**

It was determined that parents require professional support during the mathematics teaching process. Additionally, mothers perceive school as a more effective learning environment than home for teaching mathematics, value school-family cooperation, and believe that peer interaction plays a supportive role.

## 1 Introduction

Autism spectrum disorder (ASD) is becoming increasingly prevalent, with recent data indicating that approximately one in every 36 children is diagnosed with ASD (Maenner et al., 2023). This has significantly increased interest in educational research on individuals with ASD. Research has largely focused on the development of communication (Kizir, 2021; Plavnick and Ferreri, 2012; Prelock et al., 2011) and social skills (Wang and Spillane, 2009), whereas studies on academic skills have focused primarily on reading and writing (Clark and Green, 2004; Delano, 2007; Randi et al., 2010). With respect to research in mathematics, the literature primarily addresses functional life skills, such as counting and purchasing (Cihak and Grim, 2008; Denny and Test, 1995). However, the acquisition of basic academic mathematical skills, such as addition and multiplication, at an early age forms the cognitive foundation for mathematical development in later years for children with ASD (Cihak and Foust, 2008; Jordan et al., 2007).

The literature reveals that early mathematical experiences have a critical effect on children’s subsequent academic success and cognitive development. Early childhood mathematics education covers the domains of numbers, operations, algebra, geometry, measurement, and data (National Council of Teachers of Mathematics (NCTM), 2000). These skills are supported not only in school but also through mathematical interactions at home. Research shows that the home math environment enhances mathematical thinking in both typically developing children and those with developmental differences (Hurlbutt, 2011; LeFevre et al., 2009; Skwarchuk et al., 2014; Urban et al., 2025). Although the home environment allows for the acquisition of skills such as counting, patterning, and spatial relations through daily life, the process for children with ASD requires a more flexible and individualized approach. Furthermore, early mathematical development should not be limited to structured instruction alone; it should also be supported through natural and interaction-based experiences in the home environment.

This approach contributes to the generalization and maintenance of mathematical skills. Consistent with this view, Tok and Berk (2014) reported that teachers teaching mathematics to children with autism frequently rely on concrete materials and repeated practice and emphasize the importance of family support in acquiring mathematical skills. Accordingly, families should actively participate in the process of teaching math to children with ASD (Barnett and Cleary, 2015; King et al., 2016). Findings on mathematical skills in individuals with autism are limited and inconsistent. Some studies have reported strong performance in calculation and pattern recognition, whereas others have revealed difficulties in reasoning and problem solving (Chiang and Lin, 2007; Dowker, 2020). This arises from the heterogeneous structure of ASDs and individual differences. Indeed, autistic children may be relatively strong in procedural skills but may experience difficulties in reasoning and transferring skills to real-life situations (Bullen et al., 2020; Estes et al., 2011; Kasap and Ergenekon, 2017; McIntyre et al., 2017).

This finding demonstrates that learning is related not only to cognitive factors but also to environmental factors, highlighting the importance of parent–child interactions and concrete experiences at home (Akıncı-Coşgun and Tezel-Şahin, 2022). Therefore, studies examining learning opportunities at home are gaining importance in understanding the mathematical development of children with autism.

### 1.1 Mathematics in the home environment

Recent studies have shown that the home learning environment is a significant determinant of differences in children’s academic and social development (Lobo et al., 2025; O'Reilly et al., 2026; Rose et al., 2018; Tamis-LeMonda et al., 2019). Children first acquire knowledge, skills, and concepts within the family environment; in this process, the development of thinking skills is directly related to the quality of the environmental conditions provided by the family (Kilpatrick et al., 2001; Perry and Dockett, 2005).

The home learning environment focuses on four fundamental realities of family life: (1) the essential functions that parents perform on behalf of their children, (2) the deep attachment that most parents feel toward their children, (3) the central role of space in organizing family life, and (4) the power of context in shaping daily activities (Bradley, 2006). Studies have shown that the general educational processes of the home learning environment operate independently of structural characteristics and parents’ educational beliefs, whereas domain-specific educational processes are more strongly associated with these structural features and parental beliefs. Therefore, models of the home learning environment should incorporate different domains of stimulation as parallel constructs (Kluczniok et al., 2013).

With the development of the home numeracy model (Skwarchuk et al., 2014), the measurement of the home learning environment has become more detailed (Manolitsis et al., 2013). According to this model, activities are categorized into two groups: formal, which includes direct instruction, and informal, which encompasses indirect learning through interactions such as play and book reading (Skwarchuk et al., 2014). Many studies have demonstrated that these interactions support early literacy and numeracy development and predict academic achievement (Daucourt et al., 2021; Hart et al., 2016; Nelson et al., 2024; Niklas et al., 2016; Torppa et al., 2025; Zippert and Rittle-Johnson, 2020;). Additionally, there are positive relationships between the numerical support provided by parents and children’s early mathematical knowledge (Akıncı-Coşgun and Tezel-Şahin, 2022; Daucourt et al., 2021; Mutaf-Yıldız et al., 2020). These findings indicate that interactions within the home environment support learning and that this process is shaped by broader social interactions and environmental systems (Bronfenbrenner, 1979; Vygotsky, 1978).

However, parental math anxiety is related to children’s mathematics achievement, and this relationship becomes more pronounced as parent–child interaction increases (Soni and Kumari, 2017). On the other hand, it has been determined that parents with positive beliefs about mathematics engage in more math-related activities with their children, which supports the development of their children’s skills (Douglas and Rittle-Johnson, 2024; Silver et al., 2024). In this respect, the quality of mathematical interactions at home and parental attitudes are key determinants of children’s mathematical development.

### 1.2 Opportunities for children with autism: home math learning environment

The quality of the learning environment is correlated with children’s academic outcomes in later years (Conner and Cross, 2003; Melhuish et al., 2008). For children with autism spectrum disorder (ASD), the home environment is more easily individualized to meet each child’s unique cognitive and developmental needs and provide a learning space (Koegel et al., 2002; Sari, 2014). For children with ASD who face difficulties, especially in structured school environments, the home provides a critical and flexible context that supports the development of early numerical skills (Piller and Pfeiffer, 2016).

The home learning environment not only is a physical space but also encompasses the quality of mother–child interactions and family relationships. This environment influences children’s learning potential (Mermelshtine, 2017). According to Mostafa (2014), studies on ASD have focused mostly on the school environment, whereas research on the family environment remains limited. Few studies have directly examined the effects of the home environment on children with autism (Ruiz-Rodrigo et al., 2023). Most autism research focuses on school-aged children (Mostafa, 2014). Given the variability in children’s math performance (Hojnoski et al., 2023), it is important for institutions to support home-based math interventions (National Association for the Education of Young Children, 2010).

Drawing on the home numeracy model (Skwarchuk et al., 2014) and sociocultural and ecological perspectives on learning (Bronfenbrenner, 1979; Vygotsky, 1978), the present study conceptualizes early mathematics learning at home as a dynamic process shaped by the interaction between parental, child-related, and contextual factors. Within this framework, mothers’ beliefs about mathematics, mathematics anxiety, perceived competence and educational practices may influence the frequency, quality and form of the mathematical opportunities they provide at home (Douglas and Rittle-Johnson, 2024; Silver et al., 2024; Soni and Kumari, 2017). These opportunities may involve formal activities based on direct instruction as well as informal mathematical experiences embedded in play, routines, book reading and everyday family interaction (LeFevre et al., 2009; Skwarchuk et al., 2014). Therefore, mothers’ experiences are important because they reveal how parental beliefs, knowledge, available resources, and daily family practices are translated into concrete opportunities for children’s early mathematics learning.

For children with ASD, home mathematics interactions may be shaped by individual differences in communication, attention, reasoning, and the generalization of acquired skills. Although some autistic children demonstrate relative strengths in calculation or pattern recognition, others experience difficulties in reasoning, problem solving, and transferring mathematical knowledge to everyday situations (Bullen et al., 2020; Chiang and Lin, 2007; Dowker, 2020; Estes et al., 2011; McIntyre et al., 2017). Therefore, compared with typically developing children, their home numeracy environment may require greater individualization, repetition, concrete support, flexibility, and responsiveness (Piller and Pfeiffer, 2016; Tok and Berk, 2014). Accordingly, early mathematics learning at home emerges through interactions among mothers’ perceptions and practices, autism-specific characteristics, and children’s individual strengths and interests.

Despite these interrelated processes, existing research has not adequately explained how mothers interpret and organize home mathematics experiences in response to the autism-specific characteristics, strengths, and interests of their children.

### 1.3 Literature gap and the present study

Although parental and professional perceptions of children with ASD have been extensively discussed in the literature, studies examining the views and experiences of mothers of children with ASD concerning mathematics are limited (Josilowski, 2017; Urban et al., 2025). Notably, most research on home mathematics environments focuses on typically developing children (Gürgah Oğul and Aktaş Arnas, 2022; Nelson et al., 2024; Susperreguy et al., 2025; Varnell et al., 2025). However, studies specifically focusing on home-based mathematics interactions between mothers and their autistic children in Türkiye are limited. This study aims to make a unique contribution to the literature.

Accordingly, the present study aims to fill this gap in the literature by developing a deeper understanding of home-based mathematics interactions and examining the reflections of mathematical learning processes at home from the perspective of mothers of children with ASD. The findings also contribute to early childhood education practices and the development of parent support programs.

However, recent systematic reviews on teaching mathematics to individuals with intellectual disabilities have revealed that effective interventions are largely based on explicit, direct, and systematic instructional approaches (Schnepel and Aunio, 2022). These approaches appear to rely more on teacher-centered and structured processes. However, this finding indicates that mathematics teaching is predominantly addressed through school- and expert-based processes, highlighting a significant gap concerning the role of parents—especially mother–child interactions—in these processes. In this context, the present study aims to address this gap in the literature by focusing on how mothers support their children’s mathematical skills in daily life and present a more holistic perspective on early mathematics learning.

Additionally, there is limited direct empirical evidence on the relationship between early mathematics skills and cognitive development in preschool-aged autistic children; most existing studies focus on school-aged children (Bernal-Ruiz and Cerda, 2024; Zacharov et al., 2021). Similarly, most research examining person–environment interactions in the context of ASD centers on the school environment, whereas studies related to the family environment are limited (Mostafa, 2014). In this context, the fact that the present study focuses on early childhood—a younger age group—represents a unique contribution to addressing this gap in the literature on the subject.

Finally, families and teachers are significant determinants of anxiety, fear, and perceptions of failure that arise during the process of learning mathematics. In this context, understanding the experiences of mothers of children diagnosed with autism regarding their children’s mathematical learning processes is critical for developing strategies to reduce negative learning experiences and support children in understanding their environment in a more meaningful and functional manner. Early mathematical experiences are important not only for children’s immediate learning but also for their subsequent academic achievement and educational development (Duncan et al., 2007).

In summary, this study makes a unique contribution to the literature. First, by focusing on home-based mathematics interactions between mothers and autistic children in Turkey, it addresses a limited area of research in the literature. Additionally, by not restricting mathematics learning to school-based processes and examining natural and interaction-based experiences in the home environment from the mothers’ perspective, it presents a more holistic framework. Finally, by focusing on the early childhood period, this study fills a significant gap in the literature concerning this age group, which is often overlooked.

### 1.4 Purpose of the study

This study aimed to examine the mathematics learning processes of children with ASD in the home environment from the mothers’ perspective. Accordingly, this study aims to reveal mothers’ perceptions and experiences regarding mathematics instruction, how mother–child mathematics interactions take place in the home environment, and the quality of these processes. In addition, this study investigates how autism-specific characteristics are reflected in children’s mathematics learning processes and how children’s individual interests are integrated into mathematical interactions. In this respect, this study aims to develop a holistic understanding of home-based mathematics learning processes for children with autism and contributes to the limited literature on this subject.

### 1.5 Research questions

1. What are mothers’ perceptions of and experiences with teaching mathematics to their children?
2. How do mother–child mathematical interactions take place in the home environment?
3. How are autism-specific characteristics reflected in children’s mathematical-learning processes?
4. How are children’s individual interests integrated into mathematical interactions?

## 2 Method

### 2.1 Research design

This study used a qualitative research approach to examine mothers’ experiences and perceptions of the processes of learning and teaching mathematics at home for children with autism. The main reason for choosing a qualitative research approach was to thoroughly reveal mothers’ experiences with mathematics instruction, the nature of mathematical interactions occurring in the home environment, and how children’s individual characteristics and interests are reflected in these processes. Thematic analysis was used to examine the data obtained from this study. Thematic analysis is a systematic and flexible analytical approach that allows for the identification, analysis, and interpretation of recurring patterns of meaning in qualitative data (Braun and Clarke, 2006; Braun et al., 2019). This approach was considered suitable for the purpose of the study and the research questions, as it considers the individual experiences of the mothers while also enabling the identification of common patterns and meanings emerging in participants’ narratives. In the reporting of this research, the Consolidated Criteria for Reporting Qualitative Research (COREQ) were considered (Tong et al., 2007).

### 2.2 Participants

The study was conducted with 20 mothers of children diagnosed with autism who lived in a province in southern Turkey. Purposeful criterion sampling was used to select participants, and the mothers were contacted through the Provincial Directorate of Family and Social Services. The mothers’ educational backgrounds varied significantly. Five mothers were primary school graduates, seven were secondary school graduates, six were high school graduates, one had an associate degree, and one was illiterate. The main reason for focusing exclusively on mothers in this study is that, in the context in which the research was conducted, mothers were involved primarily in the daily care and educational processes of the children, as well as in supporting learning activities at home. Therefore, mothers were considered the primary participant group capable of providing direct and detailed information about children’s mathematics-learning experiences in the home environment. However, focusing solely on mothers’ experiences limits the scope of the study and leads to a lack of representation of the diverse experiences and perspectives of fathers or other caregivers.

The ages of the participating mothers’ children ranged from 5--8 years, with a mean age of 7.1 years. According to maternal reports, the children were diagnosed with ASD by child psychiatrists, mostly between the ages of 2 and 3 years. These diagnoses were not independently verified by the researchers through clinical records or additional diagnostic assessment, and ASD severity or support-need levels were not independently assessed within the scope of the study. All the children attended preschool education institutions and received support from special education and rehabilitation centers. Although the inclusion criterion specified an age range of 3–8 years, the children who participated and met all other eligibility criteria were between 5 and 8 years of age.

The inclusion criteria for the study were as follows: having a child with an autism diagnosis aged between 3 and 8 years, living in the same household as both parents, having an active role in the child’s care and educational processes, providing voluntary consent to participate in the research, and having the ability to communicate verbally in Turkish. Additionally, the absence of another disability accompanying autism in the child was an inclusion criterion. The exclusion criteria were as follows: the presence of another disability accompanying autism in the child, the mother’s lack of voluntary participation, the mother’s inability to communicate verbally in Turkish, and the child not living with both parents.

### 2.3 Instruments

To explore the home mathematics practices and experiences of mothers with children diagnosed with autism, the researchers developed a semistructured interview protocol by reviewing the relevant literature (Urban et al., 2025). The interview protocol was designed to address the research questions and to support the reliability of the study. In this context, semistructured interview questions were prepared in Turkish for the interviews with the participants. Probe questions were added to increase the comprehensibility of the interview questions and encourage participants to provide more detailed and clear responses. The first version of the form was shared with three academics from the fields of early childhood and special education. Following expert feedback, one question was removed, and minor changes were made to three questions to ensure their semantic coherence. To ensure the clarity of the interview protocol developed within the scope of this study, pilot interviews were conducted with two individuals who met the inclusion criteria but were not part of the main study sample. After the pilot interview, some changes were made to the flow and wording of the interview questions to enhance participants’ understanding, and the final version of the form was created.

### 2.4 Data collection

This study was conducted in accordance with the approval (6/92) of the Mersin University Educational Sciences Ethics Committee. The interviews were conducted face-to-face with participant mothers at the Provincial Directorate of Family and Social Services in Turkey. The interviews were conducted by the second author and an independent researcher. Prior to the start of the interviews, the researchers introduced themselves and provided the participants with information about the purpose of the study and the interview process. The participants were informed that their identities would be kept confidential, that pseudonyms would be used instead of real names, and that the information obtained would be used solely for scientific research. Written informed consent was obtained from the mothers who voluntarily agreed to participate in the study.

The interviews were conducted in a quiet room where participants could comfortably express themselves and lasted approximately 25–30 min. During the later interviews, no substantially new codes or themes emerged. Nevertheless, the remaining scheduled interviews were completed, and these interviews served to confirm the recurrence and stability of the developing thematic structure. With the participants’ permission, all interviews were audio-recorded. All the interviews were transcribed by the second author of this paper. After transcription, the texts were recompared with the audio recordings to check for possible omissions and errors in the transcription. Prior to the analysis, each participant was assigned a code, and the interview transcripts were organized into separate Word documents using these codes. The analysis was conducted using these documents.

The interviews were conducted in Turkish, and the analyses were performed using Turkish transcripts. During the reporting of the findings, the participant quotations included in the article were translated into English by a professional translation service. To ensure the accuracy of the translations and preservation of the meaning of the participants’ statements, the English translations were reviewed by the researchers in comparison with the original Turkish texts. Where necessary, revisions were made to the translations to more accurately reflect the meaning and context of the participants’ statements.

### 2.5 Data analysis

The data obtained from the interviews conducted within the scope of the study were analyzed on the basis of the six-phase thematic analysis process proposed by Braun and Clarke (2006). In the analysis process, the interview recordings were first transcribed, and the researchers read the interview transcripts several times to gain a comprehensive understanding of the data. In this phase, attention was focused on expressions related to mothers’ experiences with mathematics instruction at home, mother–child mathematical interactions, children’s autism-specific characteristics and individual interests. In the second phase, the interview texts were systematically examined, and meaningful data segments related to the research questions were coded.

During the coding process, care was taken to preserve the meaning of the participants’ statements and to generate codes directly grounded in the data. The codes related to each other in terms of content and meaning were subsequently grouped to form possible themes and subthemes for the analysis. In the next phase, the themes that had been created were reviewed again, considering both the relevant coded data segments and the data set as a whole. In this process, attention was given to ensuring that the themes formed a meaningful whole internally and were conceptually distinct. When necessary, some codes were re-evaluated, similar themes were merged, and the scope of the themes was reorganized. In the further stages of the analysis, the content of each theme and subtheme was clarified and named to reflect its relationship with the research questions. In the final phase, the identified themes were reported, supported by direct quotations that reflected the participants’ experiences.

The coding process involved the first and second researchers. The researchers independently examined the interview data and then compared the codes and thematic structures they had developed. Codes and themes that were evaluated differently were jointly reconsidered; during these evaluations, participants’ relevant statements were revisited, and the extent to which the codes and themes represented the data were discussed. The themes and subthemes that emerged from the analysis are presented in tables, and direct quotations from participants’ statements are included in the interpretation of the findings.

### 2.6 Trustworthiness, credibility and researcher positionality

Lincoln and Guba’s (1985) criteria of credibility, dependability, confirmability, and transferability were considered. To ensure credibility, a semistructured interview form was prepared in accordance with the relevant literature, expert opinions were sought during the development of the form, and pilot interviews were conducted before the main study. In addition, to make the connection between the findings and participants’ experiences visible, the themes and subthemes were supported directly by participant statements. To support dependability during the analysis process, the interview data were independently coded by the first and second researchers, and the codes generated by each researcher were then compared. The degree of agreement between the codings was calculated via the formula proposed by Miles and Huberman (1994), with the intercoder agreement determined to be 86%.

The researchers worked together on codes that were evaluated differently and revisited the relevant sections of the data to discuss discrepancies. In this process, the aim was not to eliminate different interpretations but to reevaluate the relationship between the codes and the data and examine whether the thematic structure sufficiently represented the dataset. To support confirmability, the stages followed during data collection and analysis were recorded in detail, and an audit trail of the analysis process was created. The researchers’ evaluations regarding the development of codes, the formation of themes, and the review of the thematic structure were documented throughout the process. To support transferability, the context in which the study was conducted, participant characteristics, sampling criteria, data collection process, and findings are described in detail. In addition, to strengthen the credibility of the research, a peer debriefing was conducted.

The codes, themes, and interpretations that emerged during the analysis process were critically examined and discussed with the research team by a third researcher who was not involved in the initial coding process. This process allowed us to question the researchers’ analytic assumptions, evaluate alternative interpretations, and assess whether the thematic structure adequately reflected the participants’ experiences. In qualitative research, a reflexive approach was adopted throughout the study, considering the researcher’s position in the knowledge production process. The researchers were aware that their academic and professional experiences could influence the interpretation of data. Therefore, throughout the stages of data collection, coding, theme formation, and interpretation of the findings, the researchers’ assumptions and possible influences were regularly evaluated.

The aim here was not to assume that the researcher is entirely objective but rather to make the researcher’s position in the analysis process explicit and to conduct the interpretation process as transparently as possible. The first author, an associate professor with experience in early childhood education, home mathematics environments, and qualitative research, contributed to the study’s design, preparation of the interview questions, data analysis, and development of the findings and discussion. The second author, drawing on experience in family education during early childhood and qualitative research, participated in the development of the data collection tool, conducted interviews, transcribed the data, coded, reviewed themes, and contributed to the data analysis and discussion process. The interviews were conducted by the second author and an independent researcher. The third author, who has expertise in special education and qualitative research, contributed to the research design, data analysis, and interpretation of the findings. There were no prior relationships between the researchers and the participants.

## 3 Findings

This framework conceptualizes home math interactions as a multidimensional process shaped by mothers’ perceptions and experiences, the nature of mother–child interactions, autism-specific characteristics, and the integration of children’s individual interests. Early mathematics learning in the home is not only a cognitive process but also socially and contextually constructed, emerging through dynamic interactions between parental beliefs, everyday practices, and child-specific factors.

### 3.1 Mothers’ perceptions and experiences in mathematics teaching

On the basis of the mothers’ responses, data concerning their perceptions and experiences related to teaching mathematics were analyzed. Four themes emerged from the analysis. These are “Self-Efficacy Perception, Concerns, the Need for Expert Support, and Evaluations Regarding the Learning Environment.” The themes and their subthemes are presented in Table 1.

**Table 1:** Themes and subthemes related to mothers’ perceptions and experiences in mathematics teaching.

Themes	Subthemes
Perceived self-efficacy	Lack of mathematical knowledge Lack of teaching knowledge Insufficient time
Concerns	The child’s inability to learn Lack of attention The abstract nature of mathematics Lack of confidence in explaining skills
Need for expert support	Expectation of teacher support Special education needs Rehabilitation centers
Evaluations regarding the learning environment	A more effective perception of the school School-family cooperation Peer influence Limitations of the home environment

Within the theme of self-efficacy perception, the ways in which parents assess themselves regarding mathematics instruction have been shaped by the subthemes of lack of mathematical knowledge, lack of pedagogical knowledge, and insufficient time. Indeed, while some parents highlighted their lack of mathematical knowledge with statements such as “*My math is not very good, so I struggle when explaining it.* (T1),” they also express their shortcomings in instructional knowledge with remarks such as “*I do not know how to impart my knowledge or teach my child.”* (T14)

Furthermore, a mother’s statement, “*I come home late. I get tired during the day. I cannot dedicate time after I get home.*” (T3) shows that a lack of time is a significant limitation. Despite these conditions, some parents do not see themselves as entirely inadequate and have a relative sense of competence, such as *“I explain as best as I can at different times of the day, I stop if I’m working, but I cannot say that I’m very good at it.”* (T5)

Within the theme of anxieties, parents’ concerns about the mathematics teaching process have been shaped by subthemes such as the child’s inability to learn, lack of attention, the abstract nature of mathematics, and lack of confidence in their own explanatory skills.

Mothers also stated that their children have short attention spans and are reluctant to learn.

*“I want to do activities with my child. However, shortly after starting the activity, his attention wanders, and he does not listen to what I am saying. Even if I play games to get his attention, it does not work.”* (T13)

Under the theme of the need for expert support, parents’ search for support has been shaped by subthemes such as expectations for teacher support, special education needs, and the use of rehabilitation centers. One mother stated her concerns about her child’s learning process as follows: “*I worry that she will learn slowly. Sometimes I have to repeat a simple thing repeatedly. She understands what I say very late, and she progresses very slowly in school* (T9).” Another mother expressed it as follows: “*My child generally cannot focus, gets bored quickly, and cannot concentrate. At times, it is as if she does not hear me at all* (T12).” The findings point to problems with mothers’ attention span. These findings show that parents approach the mathematics teaching process with various concerns not only at the cognitive level but also at the emotional level.

With respect to perceptions of the learning environment, parents’ evaluations of learning environments are shaped by subthemes such as viewing the school as more effective, school-family cooperation, peer influence, and the limitations of the home environment.

### 3.2 Characteristics of mother–child mathematics interactions

Data on the characteristics of mother–child mathematics interactions, obtained on the basis of mothers’ responses, were analyzed. As a result of the analysis, five themes emerged. These include “Matching and Classification, Number, Operation and Time, Comparison and Measurement, Geometric Shapes and Spatial Concepts, and Pattern.” Information regarding these themes and subthemes is provided in the Table 2.

**Table 2:** Themes and subthemes related to mother–child mathematics interactions.

Themes	Subthemes
Matching and classification	Matching activities
	Classification activities
Number, operation, and time	Counting with daily/tangible objects and with fingers, rhythmic counting (e.g., beads, beans, toys)
	The concept of hour and time
	Performing simple operations (e.g., adding concrete objects, watching videos related to counting)
Comparison and measurement	Comparing/measuring objects
Geometric shapes and spatial concepts	Geometric shape recognition (e.g., triangle, square, etc.)
	Spatial expressions (e.g., above-below, inside-outside)
Pattern	Pattern creation

Research findings reveal that mothers largely support mathematical interactions with their children through daily life practices and concrete materials. The findings related to the matching and classifying themes show that these interactions are especially conducted via visual materials.

*“We do matching with picture cards. We group the balls by color. He likes some colors more; for instance, he chooses the red balls first, then continues with the blue ones.”* (T4)

*In terms of number, operations, and time, mothers mostly engage in counting with concrete objects and rhythmic counting activities within daily routines. We do counting exercises at home with beads and toys.*” (T19)

The statement, “*We do counting exercises with beads and toys at home,*” shows that mathematical learning takes place within the context of daily life, whereas the statement, “*We watch videos about counting*,” indicates that they also utilize digital content.

Additionally, expressions such as.

*“We count the oranges in the picture on the worksheets. We do addition and subtraction with the pictures in the activity*.”(T6)*, “Sometimes I draw an object on the paper and ask how many there are, and sometimes I ask about the pictures in the activity books and magazines.”* T(10)

Mothers occasionally resort to structured materials to support their children’s mathematical skills. With respect to geometric shapes and spatial concepts, mothers integrated environmental stimuli into the teaching process. Notably, only one mother engaged in pattern activities. This mother’s statement, “*We make patterns and match using the cards*,” suggests that pattern activities are mostly carried out using structured materials. Findings show that mothers are very important in helping children turn to environmental stimuli to improve their mathematical skills.

### 3.3 Reflections of autism-specific characteristics on mathematics learning

Data on the reflections of autism-specific characteristics on the process of learning mathematics on the basis of mothers’ responses were analyzed. As a result of the analysis, five themes emerged. These are “Sensory Sensitivities, Attention and Motivation Difficulties, Use of Visual Supports, Child-Specific Interests, and Difficulties in Early Math Skills.” Information regarding these themes and subthemes is presented in the Table 3.

**Table 3:** Themes and subthemes related to the reflections of autism-specific characteristics on the mathematics learning process.

Themes	Subthemes
Sensory sensitivities	Auditory sensitivity (e.g., noise)
	Visual sensitivity (e.g., light)
Attention and motivation difficulties	Getting bored easily
	Leaving the activity unfinished
	Attention deficit
Use of visual support	Use of maps, posters, and charts
	Concrete materials (e.g., beans, bottle caps, etc.)
	YouTube videos
Child-specific interests	Music and Artistic Interests (e.g., song, music, dance, painting)
	Game and Toy-Based Interests (e.g., cars, toys, repairing)
	Nature and Environment-Based Interests (e.g., animals, plants, fruits, outdoors, park)
	Sports and Physical Activity Interests (e.g., horseback riding, swimming, cycling, active activities)
	Daily Life and Object-Based Interests (e.g., kitchen utensils, bottle caps, grocery items)
Difficulty in early math skills	Difficulty performing transactions
	Writing numbers in reverse
	Difficulty in understanding concepts

The table findings indicate that children’s sensitivities to auditory and visual stimuli limit their participation in mathematics activities and the duration of these activities. Indeed, one motherhighlighted this situation by saying, “*My child does not like loud noises, covers his ears, and gets scared; therefore, he cannot engage with mathematics for long periods*” T(15). To balance this, parents have been observed to use visual support (e.g., cards, posters, concrete materials), as evidenced by the statement, “W*e use cards as visual aids. Such as color cards, animal cards, and number cards”* T(8).

Children’s interests are mostly shaped around play, nature, and everyday objects, and learning is based on concrete and contextual experiences. On the basis of these findings, both sensory sensitivities and interest-based learning opportunities are decisive in the mathematics learning process; however, this potential is not sufficiently structured by mothers. An examination of these findings revealed that children experience various difficulties in developing early mathematics skills. These difficulties are particularly concentrated in performing operations, writing numbers correctly and understanding mathematical concepts. Mother’s statements revealed that children struggled to perform basic operations, occasionally wrote numbers backward, and had difficulty understanding some mathematical concepts.

### 3.4 The integration of individual interests into mathematics interactions

Research findings reveal that although some mothers state that their children have certain interests, they do not integrate these interests into the mathematics teaching process. The statements expressed by mothers show that children are interested in various areas, such as music (e.g., listening to songs, playing instruments), dance, sports (e.g., table tennis, swimming, horseback riding, using a scooter), visual activities (e.g., drawing), and specific objects (e.g., bottle caps, dolls). However, it is notable that mothers did not relate these interests to mathematical activities. For example, statements such as “*My child likes to play the saz and guitar, but we do not use this in math,”* T(6) and “*He’s interested in bottle caps, but we do not use this in math*,” T(13) indicate that there is no connection established between individual interests and the process of learning mathematics. This suggests that individual interest-based teaching opportunities, which could increase children’s motivation and make learning more meaningful, are not sufficiently utilized in the classroom.

Research findings also show that, although limited, some mothers integrate the individual interests of their children with autism into mathematical interactions. For example, the statement “*My child is interested in singing and we use this in math”* T (9) illustrates that music is linked to rhythmic counting and number learning.

“*My child loves playing with cars and repairing them. During the repair process, he asks how many cars need to be repaired, we count the wheels together, and I ask him to determine how many cars have not been repaired yet.*” (T12)

This statement demonstrates how the child’s play-based interests are transformed into problem situations that support mathematical skills such as counting, determining quantities, and problem-solving.

Additionally, some mothers associated outdoor activities and environmental stimuli with mathematical learning.

*“He likes riding a bike and listening to music; we sing songs while riding bikes and count the bikes that pass us.”* (T2)

*“He’s interested in cars and license plates; we count the cars that pass us while walking on the road. Sometimes he stops and asks what’s written on the license plate. We talk about the colors of the cars.”* (T3)

*Moreover, some mothers indicated that daily life contexts support mathematical skills such as counting and comparison.*

*“We do mathematics through objects in the supermarket, we count chocolates and candies, we put them in the shopping cart by counting them.”* (T14)

*In the context of interest in visual arts, one mother also connected drawing activities with mathematical learning.*

*“We use math while drawing. We talk about the shapes we draw, count the objects in the picture, and sometimes ask how many shapes or objects there are.”* T(11)

These findings show that some mothers can make learning more meaningful and motivating by transforming their children’s individual interests into mathematical interactions.

## 4 Discussion

Mothers’ experiences indicate that teaching mathematics at home depends not only on what parents know about mathematics but also on how confident they feel in adapting mathematical content to their child’s learning needs. The concerns expressed by the mothers therefore seem to go beyond a general lack of confidence in mathematics. In many cases, their uncertainty was related to difficulties in maintaining their child’s attention, explaining abstract concepts, and choosing appropriate teaching strategies. Similarly, previous studies have shown that parents’ mathematics anxiety, attitudes, expectations, and perceived self-efficacy are associated with both children’s mathematics performance and the mathematics-related activities provided at home (Becker et al., 2022; Mohr-Schroeder et al., 2017; Quaye and Pomeroy, 2022; Outhwaite et al., 2025; Silver and Libertus, 2022; Skwarchuk et al., 2014). The present findings add to this literature by showing that, for mothers of autistic children, confidence in supporting mathematics learning also involves being able to respond to the child’s individual characteristics. In this sense, mothers’ feelings of inadequacy may be linked less to an unwillingness to become involved and more to the limited guidance available to them. This interpretation is supported by Aydın and Cavkaytar (2020), who reported that fathers of children with autism were able to use systematic teaching and prompting strategies effectively after receiving training and feedback. Taken together, these findings suggest that parents’ confidence and instructional competence can be strengthened through appropriate professional support. Such support may help mothers provide more varied, purposeful, and developmentally appropriate mathematics experiences at home.

Mothers’ tendency to view school as a more effective setting for mathematics learning appears to reflect the differences they perceive between the resources available at school and at home. Schools offer structured instruction, specialist knowledge, appropriate materials, and opportunities for peer interaction, whereas mothers may feel that they have limited knowledge and resources for organizing similar experiences at home. However, this perception does not mean that the home environment has little educational value. Mothers’ accounts of counting objects, identifying shapes, exploring spatial relations, and noticing patterns during everyday routines show that mathematical learning was already embedded in family life, even when these activities were not always recognized as deliberate mathematics instruction. This finding is consistent with previous research showing that home mathematics experiences contribute to children’s learning and may include a wide range of formal and informal activities (Hornburg et al., 2021; Manolitsis et al., 2013; Purpura et al., 2020). These findings also support evidence that parental involvement can strengthen children’s understanding of mathematical operations and strategies (Pan et al., 2006) and that the home mathematics environment can benefit both typically developing children and children with intellectual disabilities (Charitaki, 2023). The contrast drawn by mothers between school and home may therefore reflect a difference in the degree of structure and professional support rather than a difference in the learning potential of the two environments. From this perspective, school and home should not be treated as competing learning settings. Strengthening school–family collaboration could help mothers recognize the mathematical value of everyday routines and use these opportunities more intentionally and confidently. This is particularly important for autistic children, who may benefit from encountering mathematical concepts across familiar and structured contexts.

Mothers’ frequent use of everyday objects, such as beads, beans, toys, balls, cups, spoons, and picture cards, suggests that mathematics teaching at home was largely embedded in familiar routines and concrete experiences. Existing evidence supports the hypothesis that concrete materials play a critical role in the development of young children’s early numerical skills (Akıncı-Coşgun and Tezel-Şahin, 2022; Ramani and Siegler, 2008). Concrete materials can effectively help highly disabled primary school students solve fraction and word problems (Marsh and Cooke, 1996). Some mothers also reported supporting their children’s mathematical skills with structured materials, such as worksheets, from time to time. Overall, mother–child mathematical interactions were found to be concrete, experience-based, and focused mainly on counting, whereas some mathematical areas (e.g., patterns) were less supported by the literature. Tok and Berk (2014) also reported that in the process of teaching mathematics to children with autism, teachers focused more on basic skills, emphasizing the use of concrete materials and repetition. These findings, parallel to the current research, show that mothers focus mostly on basic skills in their mathematical interactions with their children and support learning in the context of their daily lives. Although this emphasis is consistent with previous research, the limited attention given to mathematical areas such as patterns suggests that mothers may rely more readily on concepts that are visible and easily incorporated into their daily routines. This may be related to their uncertainty about how less familiar or more abstract mathematical content can be adapted to their children’s needs. Practical guidance should therefore move beyond recommending the use of concrete materials and provide families with examples of how a broader range of mathematical concepts can be addressed through everyday activities in the home.

Another striking finding from this research is that children’s auditory and visual sensitivities limit both their participation in math activities and the time they spend on them. Indeed, children with ASD can be more sensitive to environmental sensory stimuli (Mostafa, 2014; Sánchez et al., 2011). Parents try to balance this with visual support (cards, concrete materials); however, some mothers have not incorporated their children’s interests (e.g., songs and bottle caps) into math activities. At this point, special education and classroom teachers can support mothers by demonstrating how children’s preferred objects, activities, and interests can be incorporated into short mathematics activities that are appropriate for use at home (Bouck and Flanagan, 2010). In addition to these sensory barriers, mothers reported that their children experienced difficulties in performing mathematical operations and writing numbers correctly. Some mothers stated that they used their children’s individual interests (e.g., playing with or repairing cars) to support their mathematical skills. These findings suggest that children’s participation in home mathematics activities is shaped by the interactions among autism-related learning characteristics, sensory conditions, activity demands and individual interests (Geurts et al., 2009). Adapting the environment and incorporating preferred objects or activities may therefore be as important as selecting appropriate teaching materials or strategies.

Furthermore, considering that significant relationships have been found between mathematical skills and verbal, visual–spatial, and motor skills in children with intellectual disabilities (Kivirähk and Kiive, 2022) and that motor, emotional, and cognitive development areas are closely related in early childhood (Gil-Madrona et al., 2021; Honrubia Montesinos et al., 2023; Romero Martínez et al., 2018), it appears that focusing solely on cognitive processes is insufficient in early mathematics education. In this context, individualized, sensory-friendly, conceptually supportive, and holistic approaches that also include psychomotor development should be adopted, as these findings support an understanding of home mathematics learning as a process shaped by the interaction between the child’s characteristics, parental support, and the learning environment rather than by cognitive ability alone. Practically, parent support programs should help families plan short, sensory-sensitive, and interest-based activities that combine mathematical concepts with communication, movement, and everyday routines. Such an approach may increase children’s participation while also making mathematics teaching more manageable and meaningful for mothers and caregivers. Nevertheless, these interpretations reflect mothers’ perceptions of their children’s mathematics learning experiences.

The findings also indicate that professional support should be structured around the specific difficulties experienced by each family rather than being provided as general advice about helping children with mathematics. Special education professionals and teachers can first identify the child’s current mathematical skills, sensory needs, interests, and the routines in which the family is most likely to engage in learning activities. Support could then be delivered through brief parent-training sessions that include modeling, video demonstrations, guided practice, written or visual activity examples and individualized feedback. Teachers can help families connect classroom objectives with familiar home routines, whereas special education professionals can guide the adaptation of materials, prompt procedures, and reinforce strategies.

Psychologists and school counselors could contribute by addressing mathematics-related anxiety, parental confidence, and difficulties in sustaining parent–child interaction during learning activities. Rather than relying on a single training session, these interventions could include regular follow-up meetings in which professionals and parents review what has worked, identify difficulties, and adjust activities together. These findings have implications for teacher education. Preservice and in-service training programs should prepare special education and early childhood teachers to assess home numeracy opportunities, collaborate effectively with families, adapt mathematics activities to autism-specific characteristics, and demonstrate how everyday routines and children’s individual interests can support early math learning. Such training should emphasize individualized family guidance rather than relying solely on general recommendations for parental involvement.

### 4.1 Limitations and future research

This study had several limitations. First, the research was conducted in only one region of Türkiye, which has a relatively small sample size (*n* = 20). Additionally, the data were obtained solely from mothers’ self-reports via interviews. This restricts the inclusion of experiences from different regional and sociocultural settings, as well as from fathers, other caregivers, and teachers, in the study, thus affecting the transferability of the findings. The fact that participants were reached through the Provincial Directorate of Family and Social Services may also have limited the sample to families within a specific institutional referral network.

Another limitation of the study is that living with both parents in the same household was set as one of the inclusion criteria for the children. This criterion limits the representation of the experiences of mothers whose children live in single-parent or different family structures. Therefore, the findings should be interpreted in light of the family context of the mothers participating in this study. In this study, mother–child mathematics interactions in the home environment were not directly observed, and the children’s mathematical performance was not measured directly or via any standardized assessment tools. Therefore, the findings reflect mothers’ perceptions and descriptions of their children’s mathematics learning processes and interactions at home. The use of self-reported interviews may have been influenced by how participants recalled past experiences and their tendency to provide socially desirable responses.

Future research should work with more diverse participant groups from different regions and family structures and examine the perspectives of not only mothers but also fathers, other caregivers, and teachers. Furthermore, supporting interviews with different data sources, such as naturalistic observations, document analysis, and direct assessments of children’s mathematical performance, may contribute to a more comprehensive understanding of home-based mathematics learning processes and strengthen the transferability of the findings.

## Data Availability

The raw data supporting the conclusions of this article will be made available by the authors, without undue reservation.
